# The nutrition and immunity (nutrIMM) study: protocol for a non-randomized, four-arm parallel-group, controlled feeding trial investigating immune function in obesity and type 2 diabetes

**DOI:** 10.3389/fnut.2023.1243359

**Published:** 2023-09-01

**Authors:** Jenneffer Rayane Braga Tibaes, Maria Inês Barreto Silva, Alexander Makarowski, Paulina Blanco Cervantes, Caroline Richard

**Affiliations:** ^1^Department of Agricultural, Food and Nutritional Science, University of Alberta, Edmonton, AB, Canada; ^2^Department of Applied Nutrition, Rio de Janeiro State University, Rio de Janeiro, Brazil; ^3^Department of Medical Microbiology and Immunology, University of Alberta, Edmonton, AB, Canada

**Keywords:** immunology, hyperglycemia, nutrition, obesity, type 2 diabetes, insulin resistance, North American diet

## Abstract

**Introduction:**

Individuals with obesity and/or type 2 diabetes are at higher risk of infection and have worse prognoses compared to healthy individuals. Several factors may influence immune responses in this population, including high adiposity, hyperglycemia, and unhealthy dietary habits. However, there is insufficient data on the independent or clustered contribution of these factors to obesity-related immune dysfunction, especially accounting for dietary intake. This study aims to establish the independent contribution of obesity and hyperglycemia to immune dysfunction independent of diet in adults with and without obesity with or without type 2 diabetes.

**Methods:**

The Nutrition and Immunity (nutrIMM) study is a single-centre, non-randomized, four-arm, parallel-group, controlled feeding trial. It will enroll adults without obesity (Lean-NG) and with obesity and three metabolic phenotypes of normoglycemia, glucose intolerance, and type 2 diabetes. Participants will be assigned to one of four groups and will consume a standard North American-type diet for 4 weeks. The primary outcomes are plasma concentration of C-reactive protein and concentration of *ex-vivo* interleukin-2 secreted upon stimulation of T cells with phytohemagglutinin.

**Discussion:**

This will be the first controlled feeding study examining the contribution of obesity, hyperglycemia, and diet on systemic inflammation, immune cell phenotype, and function in adults of both sexes. Results of this clinical trial can ultimately be used to develop personalized dietary strategies to optimize immune function in individuals with obesity with different immune and metabolic profiles.

**Clinical trial registration:**

ClinicalTrials.gov, identifier NCT04291391.

## 1. Introduction

The immune system is a highly complex network that comprises molecules, cells, organs, and tissues, with the primary function of defending the host against pathogens (1). It consists of two major branches: innate and adaptive immunity, which work together to recognize foreign invaders and mount an effective immune response (2). Several factors can affect the resistance of an individual to infection, such as genetics (3, 4), age (5, 6), sex (7, 8), physical activity (9, 10), stress (11, 12), smoking (13, 14), alcohol consumption (15, 16), medications (17, 18), nutrition (19, 20), and obesity (21, 22) as previously reviewed (23).

Obesity is known to cause chronic low-grade systemic inflammation (24), which is characterized by an increased systemic concentration of acute phase proteins and cytokines, including C-reactive protein (CRP), IL-6, IL-18, and TNF-α (25–27). There is an increased release of pro-inflammatory cytokines and chemokines from adipose tissue in the presence of obesity (28, 29). This state leads to excessive recruitment and infiltration of pro-inflammatory immune cells into adipose tissue, such as M1-like polarized macrophages (30), Th1 (31, 32) and Th17 cells (33), and cytotoxic T cells (34), while reducing the number of immune cells that have anti-inflammatory properties, such as T regulatory cells (35, 36). These changes in the immune system can contribute to the development of chronic diseases, including insulin resistance and type 2 diabetes (T2D) (37, 38).

Studies have shown that obesity and T2D are associated with an increased risk of common infections from both bacterial and viral sources (39, 40). However, the mechanisms responsible for this increased risk are not fully understood. Previous research has focused on alterations in the immune system mediated by adipose tissue in obesity and/or T2D, rather than on immune function (41, 42). Recently, it was discovered that T2D is associated with additional perturbations in immune function, independent of obesity status (43). Obese individuals with T2D exhibit impaired neutrophil function and T cell response upon stimulation compared to BMI-matched normoglycemic (NG) obese individuals, despite having more activated Th cells. However, further studies are needed to determine whether the immune function of the Obese-NG group is comparable to that of lean control healthy subjects (43).

Acute inflammatory responses to dietary challenges, such as the oral glucose tolerance test (OGTT), have been proposed as a sensitive indicator of the impact of glycemia on immune responses (23, 27, 44). The OGTT induces a rise in glucose, insulin, and a transient inflammatory response (45–50), which tends to be stronger and more extended in individuals with obese and T2D (51, 52). *In vivo* studies have demonstrated that hyperglycemia increases the expression of pro-inflammatory cytokines, such as TNF-α and IL-1β, by leukocytes (53). Furthermore, after an OGTT, T2D patients had an increased proportion of neutrophils and monocytes over time compared to healthy controls, and the monocyte-AUC correlated positively with the glucose-AUC (54). These findings suggest that glycemia plays a role in immune activation, and further investigation is needed to understand the impact of glycemia on immune responses in individuals with obesity.

Nutrition plays a crucial role in the prevention and treatment of health conditions that have an inflammatory component, such as obesity (55, 56). Saturated fatty acids (SFA) have been shown to have pro-inflammatory properties, while fiber, antioxidants, vitamins, and polyunsaturated fatty acids (PUFA), especially eicosapentaenoic acid (EPA) and docosahexaenoic acid (DHA), generally exert anti-inflammatory and/or immunosuppressive effects in the presence of chronic inflammation as previously reviewed (20). Choline is another nutrient that has been shown to be essential for optimal immune function (57), improving the T cell response to immune challenges by increasing IL-2 secretion (58, 59). These findings suggest that specific nutrients in the diet could have a beneficial effect on immune function, particularly in the context of obesity.

Despite the association between obesity, hyperglycemia/T2D, elevated systemic inflammation, and impaired immune cell response, it remains unclear whether these abnormalities are caused by excess adiposity, dysglycemia, or poor food habits associated with obesity. To dissect the impact of excess body fat and glycemia on immune function, it is crucial to account for food intake by performing controlled feeding studies in humans. Therefore, a comprehensive analysis of systemic inflammation and immune function in healthy lean individuals (Lean-NG), individuals with obesity and normoglycemia (Obese-NG), glucose intolerance (Obese-GI) or T2D (Obese-T2D) is required to understand the independent contribution of excess body fat and glycemia on immune function.

## 2. Methods

### 2.1. Study objectives and hypothesis

The study objectives are:

1. To establish the independent contribution of obesity and glycemia, independent of diet, on systemic inflammation and immune function outcomes. We hypothesize that systemic inflammation will increase and T cell function, characterized by *ex vivo* IL-2 secretion upon phytohemagglutinin (PHA) stimulation, will decrease progressively from the Lean-NG to Obese-T2D group.
2. To determine the role of glycemia on postprandial inflammatory and immune responses using an OGTT. We hypothesize that a higher glucose AUC will be associated with increased inflammatory responses, a higher proportion of activation markers expressed on immune cells, and a lower proliferation rate by PBMC upon stimulation with anti-CD3/CD28 in Lean-NG, Obese-NG, Obese-GI, and Obese-T2D individuals.
3. To explore the relationship between diet, metabolism, and immune function. We hypothesize that a higher habitual consumption of DHA and choline and lower consumption of SFA, will be associated with enhanced immune function and lower levels of systemic inflammation. Specifically, a higher proportion of DHA in the membranes of RBC will be associated with improved Th1 response (i.e., IFN-γ and TNF-α), despite similar production of IL-2 upon immune challenge, whereas systemic inflammatory markers will be lower. Higher consumption of choline in the form of phosphatidylcholine will improve IL-2 production upon T cell stimulation. Other dietary components (e.g., fiber, antioxidants, and vitamins) will be associated with enhanced immune function in individuals with and without obesity with varying levels of glycemia.

### 2.2. Outcomes

The primary outcomes of this study are the concentration of IL-2 in the supernatant of *ex vivo* stimulated cells, which is a surrogate marker of T cell proliferation, and circulating concentrations of CRP.

### 2.3. Study design

**Figure 1:**
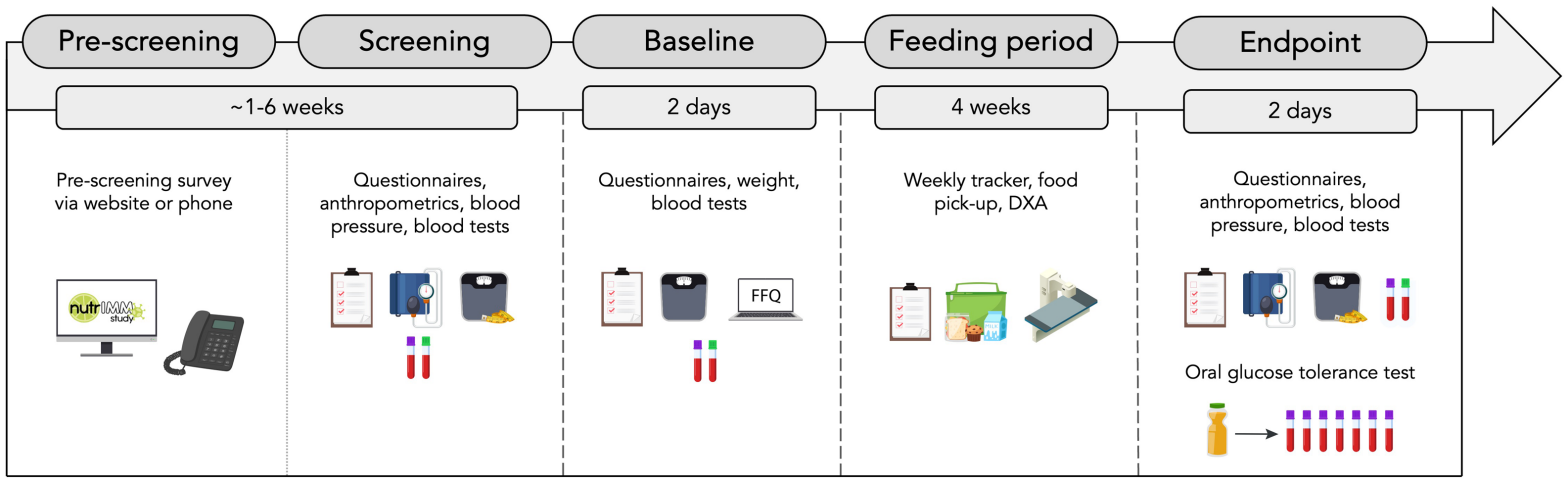
Overview of study design. FFQ, food frequency questionnaire and DXA, dual-energy X-ray absorptiometry.

The NutrIMM study is a prospective, non-randomized, four-arm, parallel-group, unicentre, controlled-feeding trial conducted at the Human Nutrition Research Unit (HNRU), University of Alberta (Edmonton, AB, Canada). A total of one-hundred and twenty-eight participants aged between 18 and 70 years will be assigned with a 1:1 male to female ratio to one of four groups (*n* = 32 per group): Lean-NG, Obese-NG, Obese-GI, Obese-T2D. All participants will be followed up for a total of 4 weeks from the date of group allocation. It will not be possible to blind participants or researchers due to the inclusion specifications of the groups. An overview of the study design is shown in Figure 1.

### 2.4. Ethical aspects

The research protocol was approved by the University of Alberta Ethics Board (Pro00085839) and follows the standards proposed by the Canadian Tri-Council Policy statement on the use of human participants in research. This protocol was developed according to the Standard Protocol Items: Recommendation for Interventional Trials (SPIRIT) (60) and the Template for Intervention Description and Replication (TIDieR) (61). All participants will sign an informed consent document approved by the Human Research Ethics Board of the University of Alberta for study participation and to optionally provide stool samples for a sub-study to investigate the role of gut microbiota on immune function (Supplementary File 1). Participants will sign an additional consent form to select if they allow their biological specimens to be stored for up to 20 years for use in ancillary studies.

### 2.5. Recruitment and enrolment of participants

#### 2.5.1. Recruitment

The trial will recruit males and females from the Edmonton Metropolitan Region via the traditional media (newspaper, radio, and TV), word of mouth, institutional websites, social media advertisements, and posters placed on notice boards at the University of Alberta, obesity clinics, and other facilities from the surrounding area of Edmonton. A website and social media accounts (Instagram and Facebook) with the study information were also developed to support recruitment.^1^ Individuals can be included in the study if they meet all inclusion criteria and no exclusion criteria. All participants will be matched by age and sex, if possible, and participants with obesity will be matched for BMI and/or waist circumference. All females in reproductive stage which are not using contraceptive methods will be tested during the same follicular phase of their menstrual cycle (day 2 to 9), to account for immunometabolic changes, considering 4 weeks the mean duration of a menstrual cycle (62, 63). Participants will be allocated to one of four groups according to specific criteria for BMI, waist circumference, blood pressure, fasting glucose, hemoglobin A1c (HbA1c), triglycerides, and high-density lipoprotein-cholesterol (HDL-C) (Table 1). The cut off points used to identify obesity, cardiometabolic risk factors, and T2D were based on the World Health Organization (64), National Cholesterol Education Program-Adults Treatment Panel III criteria for metabolic syndrome (65) and Diabetes Canada (66), respectively.

**Table 1:** Eligibility criteria per group of adults.

Criteria	Lean-NG	Obese-NG	Obese-GI	Obese-T2D
BMI (kg/m^2^)	18.5–24.9 (±0.5)	≥ 30 (±0.5)		
Waist circumference (cm)	Males <102; Females <88	Males ≥102; Females ≥88		
Fasting blood glucose (mmol/L)^a^	<5.6		5.6–6.9	≥ 7.0
HbA1c (%)	<5.5		5.5–6.4	≥ 6.5^b^
BP (SBP/DBP, mmHg)	<130/85		NR	
Triglycerides (mmol/L)	<1.7		NR	
HDL-C (mmol/L)	Males ≥1.03; Females ≥1.29		NR	

BMI, body mass index; BP, blood pressure; DBP, diastolic blood pressure; HbA1c, glycated hemoglobin; HDL-C, high density lipoprotein-cholesterol; NR, not required in it that could be normal or elevated; SBP, systolic blood pressure; T2D, type 2 diabetes.

a If participants are at the upper or lower limits for fasting glucose, HbA1c will be used for group allocation.

b Or diagnosis of type 2 diabetes and use of medication.

#### 2.5.2. Inclusion criteria

- age of 18 years to 70 years;
- body weight stable (± 3%) for at least 3 months prior to study commencement;
- BMI (1) between 18.5 and 24.9 (± 0.5) kg/m^2^ or (2) between 30 and 50 kg/m^2^ (± 0.5) kg/m^2^ or waist circumference > 88 cm or > 102 cm for females and males, respectively;
- See Table 1 for specific group allocation criteria based on glucose, HbA1c, HDL-C, and triglycerides.

#### 2.5.3. Exclusion criteria

- current or recent history cardiovascular diseases or events (e.g., ischemic, rheumatic, or congenital heart disease, stroke, peripheral vascular disease, heart failure, familial hypercholesterolemia or other monogenic dyslipidemia), use of cardiac implantable electronic devices;
- current or recent cancer, including remission, during the last 5 years;
- diseases known to affect the immune system, such as infectious, inflammatory, and autoimmune diseases or autoimmune-related or suspected conditions (e.g., T1D, systemic lupus erythematosus, inflammatory bowel disease), except for psoriasis, atopic dermatitis, and rheumatoid arthritis. Continuous use of anti-inflammatory or immunosuppressant drugs and supplements for which washout is not possible, except for medications which participants with obesity could not refrain from (e.g., baby aspirins);
- renal disorders, endocrine disorders other than T2D (e.g., acromegaly, Addison’s disease, Cushing’s disease);
- untreated or uncontrolled thyroid diseases (e.g., Hashimoto’s disease, hypothyroidism, hyperthyroidism);
- known allergy, aversion to any components of the menu, or restricted dietary patterns (e.g., gluten-free diet, vegetarianism, kosher or halal diets) for which accommodations within the menu are not possible;
- participants under titration of their medication or initiating a new treatment or HbA1c >10.5%
- women who are pregnant or plan to become pregnant during the study duration, who are lactating, who have an irregular menstrual cycle or are in perimenopause;
- regular recreational use of cannabis;
- taking part in any other intervention study that might affect the outcomes of the current study.

#### 2.5.4. Enrolment

**Figure 2:**
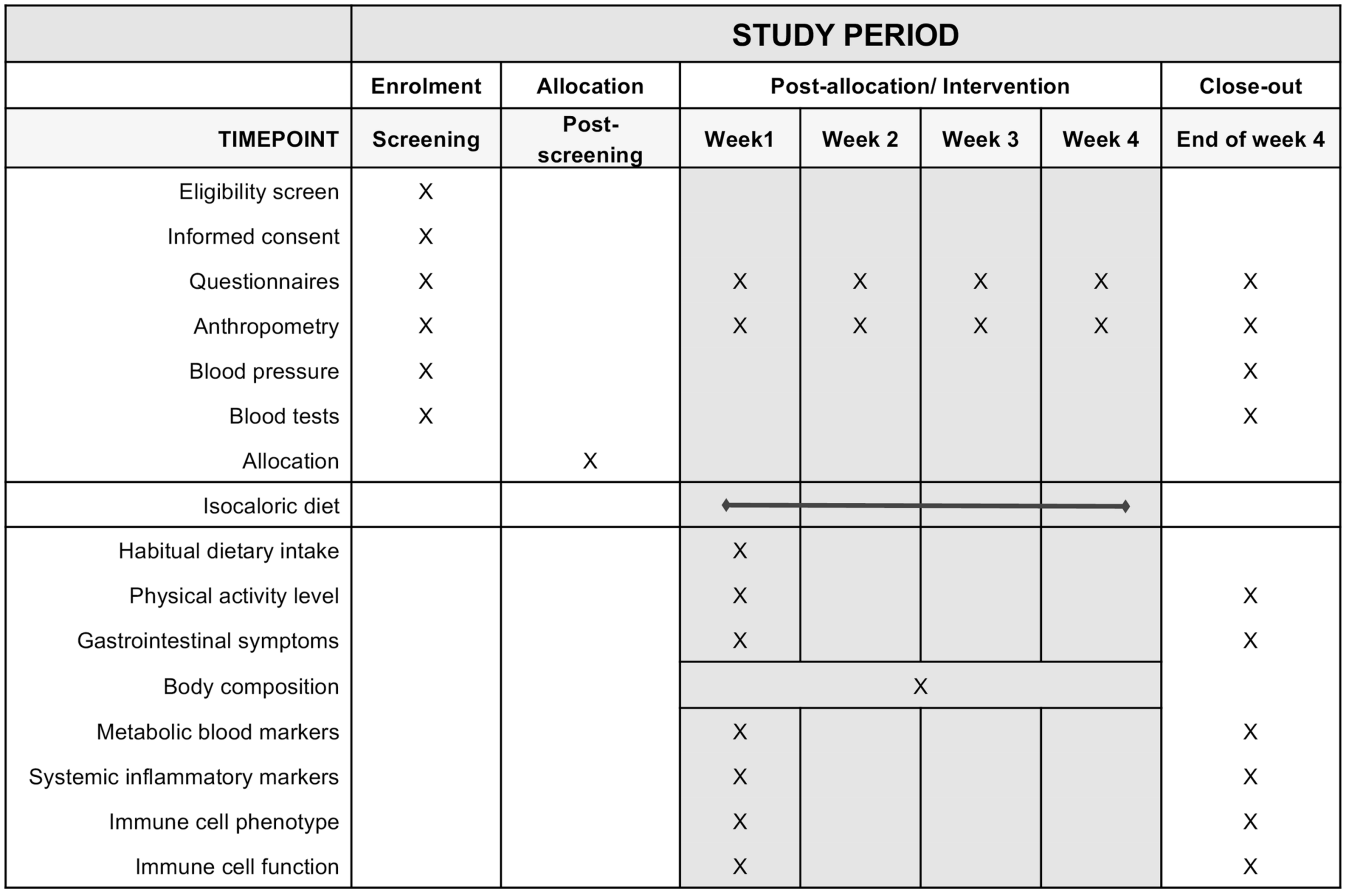
Schedule of enrolment, interventions, and assessments (SPIRIT figure).

Potential participants will complete a pre-screening and screening visit before entering the study. The pre-screening consists of a structured questionnaire, which can be completed online or through the telephone (Supplementary File 2). All pre-screening data is stored on the web-based software Research Electronic Data Capture (REDCap) (67). The study coordinator follows up with interested individuals by email and/or telephone to schedule their screening visit or let them know in case they are not eligible. At the screening visit, a trained research staff member (e.g., research coordinator, graduate students, investigators) will obtain written consent from interested individuals to confirm their eligibility by checking every inclusion and exclusion criteria. This visit includes blood tests, anthropometric measurements, and the completion of questionnaires regarding demographics, health, use of medications, and physical activity. During or after the screening visit, participants will not be enrolled in the study if they decline to participate after knowing more study details or if they violate any inclusion criteria. The enrollment period might generally vary from 3 days to 5 weeks depending on the necessity of a washout before study commencement or the availability of participants. The schedule of study visits and procedures are depicted in Figure 2.

### 2.6. Withdrawal of participants

Participants may be withdrawn from the trial either at their own request or at the discretion of the principal investigator if not compliant with the menu. Participants will be made aware via the information sheet and consent form that data collected up to the withdrawal date may still be used in the final analysis. If participants have consumed the control diet for at least 2 weeks, they will be asked if they would like to provide a blood sample before withdrawal. The reason for and date of withdrawal will be recorded on an electronic spreadsheet.

### 2.7. Intervention

#### 2.7.1. Concomitant care and pre intervention requirements

The administration of vaccines, application of botulinum toxin, use of antibiotics, supplements or other natural products, and blood donation is not permitted throughout the intervention and a four-week washout is necessary prior to study commencement. For recent surgical procedures, approximately 4 months or less, inclusion will be at the discretion of the principal investigator depending on the magnitude of the procedure. Participants should continue to take their regular medications [e.g., antilipemic (fenofibrate and atorvastatin), blood pressure-lowering (amlodipine, enalapril), antidepressants (escitalopram, bupropion, and amitriptyline), and glucose-lowering (metformin, insulin)]. Participants will be asked to refrain from performing vigorous physical activity, taking anti-inflammatory medications, and drinking alcohol 3 days prior to baseline study visits.

#### 2.7.2. Standardized diet

Participants will all consume an isocaloric standardized North American type diet for 4  weeks. All meals will be provided to participants for an optimal control of energy and nutrient intake. A seven-day cyclic menu was designed using The Food Processor Nutrition Analysis Software (v.11.0.3, ESHA Research, Salem, OR, 2015) to reflect as closely as possible current macronutrient intake averages in North America (68–70) with approximately 35% of energy as fat, 12.5% as SFA, 13% as monounsaturated fatty acids, 6% as PUFA, 48% energy as carbohydrates with a significant proportion of these coming from foods containing refined sugars, and 17% as proteins mostly from meat, meat products and dairy products. The breakfast meal represents approximately 20% of the daily energy intake whereas the lunch and dinner meals each provides 40% of daily energy intake (Supplementary File 3). Participants will be instructed to consume their meals entirely but will have the opportunity to spread out their food throughout the day. Participants will have free access to water and other non-caloric beverages during the feeding period according to their habitual consumption (or nearly calorie-free drinks, i.e., black coffee or tea). Adaptations will be made for participants with lactose intolerance, including the use of lactose-free milk, lactase, or fortified vegetable beverage (soy or rice), and for individuals with an aversion to vegetables or other meals of the menu when an adequate substitute based on nutrient composition can be provided.

#### 2.7.3. Estimate of energy requirements

Total energy expenditure of participants will be estimated using the Mifflin St. Jeor equation and metabolic equivalents (MET) to estimate physical activity level using a template spreadsheet by Gerrior et al. (71). If the desired MET was not available in the spreadsheet, they were inserted according to the 2011 Compendium of Physical Activities (72). Energy intake will then be prescribed for each participant according to their individual estimate of total energy expenditure. The energy content of the diet will be revised weekly according to body weight fluctuations throughout the intervention period. If body weight has a significant slope (i.e., increasing or decreasing) at a rate that yields a change ≥3% during the first 2 weeks, adjustments to the energy content of the diet will be made to offset changes in body weight and keep it constant for the remaining of the study period. Participants will be instructed to maintain their usual physical activity level throughout the intervention period except for the 3 days preceding blood sampling, during which they will be asked to refrain from intense physical exercise.

#### 2.7.4. Food preparation and delivery

All menu items (Supplementary File 4) will be prepared and portioned at the metabolic kitchen of the HNRU in accordance with the food safety standards and guidelines set by the Alberta Health Services. All food handlers have received training on food safety. Meals are prepared weekly and in batches. Batch cooking will be used to optimize weekly meal preparation and delivery to participants. Samples (200 g) of food cooked in batches will be saved and frozen up to 3 months after the last batch is consumed in case food safety analysis is needed. Preparation of high-risk foods (e.g., containing eggs or meat) will be temperature-controlled and recorded in a form. Meals will be prepared from Monday to Thursday and batch cooked meals are thawed overnight at 4°C on the day before portioning (Sunday–Wednesday). Meals will be placed into reusable ready to eat containers that are microwavable and dishwasher safe, sealable plastic bags, or disposable containers. All menu items are labeled, packed into reusable thermal bags, and maintained at 4°C until pick-up time. Food will be individually delivered by a trained study staff, including graduate students or other faculty members of the study team. Participants will come to the research unit to pick up their food every other day and on Friday they receive meals for Saturday, Sunday, and Monday. Participants will be instructed and will sign an agreement to store their food at 4°C within one-hour time frame and to properly re-heat it before consumption. Reusable items will be sanitized using a 200 ppm chlorine solution prepared daily (i.e., thermal bag and ice packs) or at 82^o^ C using a commercial dishwasher (Moyer Diebel Ltd. 501HT-70, Winston Salem, NC, United States) (i.e., food containers).

### 2.8. Follow-up and extra visits

Participants will be followed up to three times a week during their food-pick up. This visit takes approximately 10 min and includes body weight measurement, follow-up regarding compliance, scheduling of next visits, delivery of thermal bag containing meals and frozen reusable ice packs, and collection of reusable items (i.e., empty containers and ice packs) from participants. Additional site monitoring visits may be scheduled in case participants require additional assistance from the study team to complete the food frequency questionnaire FFQ, which is used to assess habitual dietary intake. Participants will be able to discuss any aspect of the study with the trial manager, including their blood tests, body composition, and dietary assessment results. To help retain participants on the study, they will receive emails or phone calls to support them throughout the duration of the study and will be reminded about their scheduled visits.

### 2.9. Data collection

#### 2.9.1. Compliance

The participant will complete each week a menu and health tracker (Supplementary File 5), in which they will register their food consumption, medications, and symptoms. Before the study commencement, participants will be instructed to only consume the meals provided by the study team and they will be encouraged to do so during weekly visits. Regardless, the tracker provides a space to indicate unlisted food items that will have been consumed in addition to the formulated diet. Compliance will be determined by calculating the number (and proportion) of food items consumed that were provided and those consumed in addition. Participants who cannot comply to ≥90% of the feeding protocol will be excluded from the study. Participants will also be asked to record any medications (including over the counter medications) that were taken during the study period to account for any changes in inflammation. Participants will be asked to avoid if possible, taking any anti-inflammatory medications, and use acetaminophen instead if able. Regarding gastrointestinal tolerance, participants will complete a gastrointestinal tolerance questionnaire at the beginning and end of the study, to evaluate the effect of the diet on the GI system.

#### 2.9.2. Anthropometric measures, body composition, and blood pressure

Anthropometric measurements including weight, height, waist, and hip circumference will be assessed in duplicate according to standardized procedures (73, 74). Body weight will be measured to the nearest 0.1 kg using a calibrated digital scale (Health o meter^®^ Professional Remote Display, Sunbeam Products Inc., Fla., United States). Body weight will be recorded every other day at food pick-ups throughout the feeding period. Height is measured to the nearest 0.1 cm using a 235 Heightronic Digital Stadiometer (Quick Medical, North Bend, Wash., United States). Waist and hip circumference are measured to the nearest 0.1 cm, using a measuring tape (Hoechstmass^®^, Sulbzbach, Hesse, Germany).

Body composition is assessed once during the study by dual-energy X-ray absorptiometry (DXA) using a General Electric Lunar iDXA with encore 13.60 software (General Electric Company, Madison, Wis., United States), as described (75). This scan provides compartmentalized and whole-body data on fat mass (FM), lean soft tissue (LST), and bone mineral content (BMC). The coefficients of variation of this device for FM (%), FM (g), LST (g), and BMC (g) are 1.05, 0.99, 0.37, and 0.40%, respectively. The assessment is performed by a certified technologist. All females at premenopausal who are not using contraceptive methods will perform a rapid urine human chorionic gonadotropin immunoassay to confirm non-pregnant status before scanning.

Systolic and diastolic blood pressures will be measured in the left arm after a 10 min rest in the sitting position using a calibrated automatic blood pressure monitor (Spot Vital Signs^®^, Welch Allyn^®^, Skaneateles Falls, NY, United States).

#### 2.9.3. Physical activity and habitual diet

Participants will be required to maintain their current physical activity levels throughout the study period. They will complete the self-administered International Physical Activity Questionnaire (IPAQ) – Short Form, at baseline and post-intervention. The IPAQ inquires about time spent sitting, walking (3.3 METs), and doing moderate (4.0 METs) and vigorous-intensity (8.0 METs) activities over the past seven consecutive days. All continuous scores are expressed in MET-minutes/week and used to categorize physical activity level as low, moderate, or high. The IPAQ is a reliable questionnaire, and it has been validated in different settings across multiple countries (76).

Habitual dietary intake will be assessed at baseline using the Diet History Questionnaire III (DHQIII) (77, 78). The DHQIII is a validated food frequency questionnaire and will be administered to participants before the start of the study to estimate their habitual dietary intake of the past 4 weeks. We have selected the option to inquire about the food intake of the previous month to minimize recall bias as compared to covering the previous year.

#### 2.9.4. Fasting blood collection

Blood will be sampled from participants by venipuncture at five time points, after a 9–12 h overnight fast, by a trained phlebotomist. Blood sample is collected into BD Vacutainer^®^ tubes (Becton, Dickinson and Company, Franklin Lakes, NJ, United States), with spray-coated lithium heparin and a polymer gel or spray-coated K_2_-EDTA for plasma separation.

At the screening visit (Visit 1), blood samples (~ 8.5 mL) will be sent to the Alberta Precision Labs (Edmonton, AB, Canada) immediately after collection and are analyzed for HbA1c (4 mL K_2_-EDTA tube) or fasting glucose, triglycerides, total cholesterol, and HDL-c (4.5 mL lithium heparin tube). These analyses will be repeated at the baseline visit in case it is more than 2 weeks apart from the screening visit.

At baseline (visit 2 and 3) and final visits (visit 4 and 5), one K_2_-EDTA tube (~ 4 mL) will be analyzed for complete blood count and differential and HbA1c, and one lithium heparin tube (~ 4.5 mL) will be analyzed for glucose, insulin, lipid panel, and CRP in two consecutive days, to preclude an ongoing infection (i.e., CRP >10 mg/L), by Alberta Precision Labs (as above). Four K_2_EDTA tubes (~ 24 mL) will be analyzed in-house. Samples are kept at 4°C until being centrifuged at 1811 g for 10 min at 22°C. Plasma will be aliquoted for storage at -80^o^ C for subsequent analysis. The remaining buffy coat (leukocytes and platelets) and erythrocytes are resuspended with 3 mL of 1% bovine serum albumin (BSA, Sigma-Aldrich, Co., St. Louis, MO, United States) in phosphate-buffered saline (PBS) and layered onto Ficoll-Paque (HISTOPAQUE^®^-1,077, Sigma-Aldrich, as above), then centrifuged at 1558 g for 30 min with no brake at 20°C for PBMC isolation using density centrifugation. The lymphocyte band at the gradient interface of 1% BSA in PBS and Ficoll-Paque is transferred to a 50 mL conical tube, brought to 30 mL with 1% BSA in PBS, and centrifuged at 478 g for 5 min at 4 ^o^ C to pellet PBMCs. Supernatant is discarded and PBMCs are then washed with 10 mL 1% BSA in PBS and centrifuged as previously. PBMCs are resuspended in a cryopreservation solution containing Roswell Park Memorial Institute (RPMI) 1,640 medium (Thermo Fisher Scientific, Waltham, MA, United States) supplemented with 20% (v/v) fetal calf serum (FCS, Thermo Fisher Scientific, Waltham, MA, United States), 25 mmoL/L 4-(2-hydroxyethyl)-1-piperazineethanesulfonic acid (HEPES, Corning, Manassas, VA, United States), 2.5 μmol/L 2-mercaptoethanol (Thermo Fisher Scientific, Grand Island, NY, United States), and 1% (v/v) antibiotic/antimycotic solution (AB/AM, Sigma-Aldrich, as above) with 10% (v/v) dimethyl sulfoxide (DMSO, MP Biomedicals, Solon, OH, United States) added before use. PBMCs are then aliquoted to count using the trypan blue (diluted 1:1 with ddH_2_O, Corning, as above) membrane dye exclusion method using a 1:1 ratio of cell suspension to trypan blue. The remaining cell suspension is transferred to cryovials in 1 mL aliquots and immediately frozen at -80^o^ C using a freezing container (Mr. Frosty™ Cryo 1°C, Nalgene^®^, Rochester, NY, United States). PBMCs are transferred to liquid nitrogen the next day for long-term storage. The RBC fraction is washed with 5 mL 0.9% saline and centrifuged at 453 g for 5 min at 4°C. Supernatant is discarded and RBCs are washed and centrifuged as previously. RBCs are lysed by bringing to 3 mL with double-distilled water (ddH_2_O), then stored at -80^o^ C for subsequent lipid analysis.

#### 2.9.5. Oral glucose tolerance test

The OGTT will be conducted once at the end of the feeding protocol. After a 9–12 h fast, an intravenous (IV) catheter will be inserted into a forearm vein for blood sampling by an experienced licensed practical nurse. After the IV catheter will be inserted, it will be flushed with saline before the first collection, and following each blood draw to ensure patency and rinse blood out of the catheter. Prior to each blood draw, a discard tube will be filled to a volume of at least 2 mL to clear the saline of the IV line. Blood samples will be collected into BD Vacutainer^®^ tubes (Becton, Dickinson and Company, as above), with spray-coated K_2_-EDTA for plasma separation. After the first blood collection (time 0), participants will be given up to 5 min to drink a beverage (296 mL) containing 75 g of dextrose (Trutol^®^ Glucose Tolerance Test Beverage). Then, blood samples will be taken every 30 min (±5 min) up to 180 min. For each time-point, ~ 6 mL of blood will be drawn. The IV site will be regularly monitored for phlebitis according to the Visual Infusion Phlebitis Score. During the test, participants will be allowed to drink water *ad libitum*. Plasma separation and PBMC isolation will be carried as described for fasting samples.

#### 2.9.6. Stool collection

Fecal samples are collected by participants at home or on-site before the start of the study diet and at the end of the study. The collection kit, which includes a collection device (FecesCatcher, TAG HEMI) and a fecal collection tube (DNA/RNA Shield™, Zymo Research Corp, United States) will be provided in a sealed plastic bag along with the instructions for proper handling of samples. Fecal samples will be kept at room temperature until brought to the research unit and will be stored at -80^o^ C for subsequent analysis.

#### 2.9.7. Biochemical analysis

##### 2.9.7.1. Cardiometabolic risk factors/ systemic inflammation

Biochemical analysis performed by Alberta Precision Labs includes (1) enzymatic colorimetric assays for triglycerides, total cholesterol, and HDL-C; (2) UV testing using an enzymatic reference method with hexokinase to quantity glucose; (3) an immunoturbidimetric assay for quantification of CRP, all using an automated photometric analyzer (Roche Cobas c503); (4) an electrochemiluminescence immunoassay to determine insulin using an automated immunology analyzer (Roche Cobas e801); and (5) a turbidimetric inhibition immunoassay for hemolyzed whole blood to determine HbA1c using an analyzer designed specifically for HbA1c quantification (Roche Cobas c513). Low-density lipoprotein-cholesterol (LDL-C) is calculated using the following equation: Totalcholesterol−HDLc−(Triglycerides÷2.2). Non-HDL-C is derived from the calculation of total cholesterol minus HDL-C. The Homeostatic Model Assessment for Insulin Resistance (HOMA-IR) index will be calculated using the equation: fasting insulin (microU/L) × fasting glucose (nmol/L)/22.5 (79).

Fasting and post-OGTT levels of circulating cytokines, chemokines, and soluble adhesion molecules will be measured using multiplex assay kits according to the instructions of the manufacturer. All samples will be run in batches with all timepoints per participant on the same plate to minimize variation. Post-OGTT plasma glucose will be measured using a clinical chemistry analyzer (Abbott ARCHITECT c4000, Canon Medical Systems Corporation, Otawara, Tochigi, Japan). The machine is calibrated and compared to controls based off manufacturer recommendations with a CV <5%.

##### 2.9.7.2. Immune cell phenotype analysis

Fasting complete blood count and differential will be analyzed in whole blood by fluorescent flow cytometry using an automated hematology analyzer (Sysmex XN10) by Alberta Precision Labs. Immune cell subsets from fresh whole blood are identified in-house by direct immunofluorescence assay. Briefly, 96-well V-bottom plates (Costar^®^, Kennebunk, ME, United States) are pre-conditioned with 200 μL 5% FCS in PBS with AB/AM (IF buffer) for at least 30 min before adding 100 μL of whole blood. The RBCs are lysed using 200 μL of 1X RBC lysis buffer (BioLegend, San Diego, CA, United States). After a 15 min incubation at room temperature, the plate is centrifuged at 402 g for 10 min at 10°C to pellet RBCs. Supernatant is discarded using needle aspiration and RBCs are lysed as previously, incubated for 5–10 min, and plate is centrifuged. If RBC are still evident, a third lyse (5–10 min) will be performed. After RBC lysis, supernatant is discarded and immune cells are washed twice with 200 μL IF buffer and incubated for 30–60 min at 4°C in the dark with a mix of fluorophore conjugated antibodies (except FOXP3) to characterize immune cell phenotypes. The antibodies conjugates, staining reagents, and the corresponding multicolour flow cytometry panels designed to identify immune cell subsets and activation markers [(1) T reg, (2) T cell A, (3) T cell B, (4) B cells, (5) Monocytes, (6) DCs, (7) NK, and (8) T helper] are shown in Supplementary Table S1. After incubation, cells from panels 2–8 are washed twice as previously. Cells are resuspended in 1% paraformaldehyde in PBS for at least 1 hour to fix cells before being transferred to FACS tubes containing 100 μL of IF buffer and stored away from light at 4°C until acquiring. Following the first wash, the Treg cells (panel 1) are resuspended in FoxP3 Fix/Perm solution (Fix/Perm Buffer Set, BioLegend, as above) diluted in PBS, transferred to FACS tubes, and incubated for 20 min at room temperature in the dark for further intracellular FoxP3 staining. Tubes are centrifuged at 428 g for 2 min at 10°C to pellet cells and supernatant is discarded. Cells are washed with FoxP3 Perm buffer solution diluted in PBS and pelleted. After discarding supernatant, cells are resuspended in diluted FoxP3 Perm buffer and incubated, in the dark, at room temperature for 15 min. Cells are pelleted and after discarding supernatant, they are resuspended in 20 μL of anti-FoxP3 antibody mix (diluted with Perm buffer), and incubated at room temperature, in the dark, for 30 min. Cells are washed twice in IF buffer as previously. Cells are resuspended in 300 μL of IF buffer and stored as previously described.

Negative and positive gates are determined using compensation beads (AbC™ Total Antibody Compensation Bead Kit, Thermo Fisher Scientific, as above) to control for fluorochrome spillover. Tandem-specific compensations are employed to account for differences in compensation lot to lot/ vial to vial. Briefly, we label microtubes and FACS tube for each antibody (*n* = 13). Antibodies are diluted in 1:100 μL [BV510, BV421, PerCP, APC, FITC, CD28 (BV711), HLA-DR (BV711)] or 1:200 μL [PE, CD45RO (BV711), CD196 (BV711), CD86 (PECy7), CD185 (PECy7), CD192 (PECy7)] using IF buffer. One drop of positive and one drop of negative beads is added to each FACS tube. 50 μL of previously diluted antibodies are transferred to FACS tubes and mixed with beads. Tubes are incubated at 4°C in the dark for 30 min. IF buffer (200 μL) is added and tubes are centrifuged at 428 g for 2 min at 4°C; 2 min. Supernatant is discarded and wash steps are repeated. Compensations are resuspended in 300–600 μL IF buffer and stored at 4°C await from light. All samples, including compensations, will be acquired within 72 h using a BD LSR-Fortessa X-20 flow cytometer (BD Biosciences, San Jose, CA) and analyzed according to the relative fluorescence intensity using a platform for single-cell flow cytometry analysis (FlowJo 10.8.1, Becton Dickinson & Company). The flow cytometer machine goes through rigorous quality control, including periodical calibration, and is located at the Faculty of Medicine and Dentistry Flow Core at the University of Alberta, which is a recognized laboratory by the International Society for Advancement of Cytometry. Quality control of acquired samples will also be made using a method that adopts algorithms for the detection of anomalous data (flowAI) as previously described (80). Data is processed automatically, with the call of an R function, by optimizing flow rate, signal acquisition, and dynamic range (80). The population of monocytes and lymphocytes (i.e., PMBCs) will be the starting point gate of all immune subsets, which will be determined using established gates based on morphological characteristics of forward and side scatter. Fluorescence minus one (FMO) control will be used to establish positive staining when needed. All analyses are carried out by one individual and gates are reviewed by another to minimize interobserver variability.

##### 2.9.7.3. Immune cell recovery

PBMC cryovials previously stored in liquid nitrogen will be rapidly thawed in a 37°C water bath for 5 min. The cryovials are dried and wiped with 70% ethanol before opening, then PBMCs are transferred to a 9 mL aliquot of 10% FCS RPMI prewarmed at 37°C. PBMCs are spun down and rinsed with 10 mL of 10% FCS RPMI to remove DMSO. PBMCs are resuspended in 5 mL of 10% FCS RPMI and transferred to 12 mL cell culture tubes to rest overnight at 37°C 5% CO_2_. (Forma™ Series II Water Jacket CO_2_ incubator 3,110, Thermo Fisher Scientific, Asheville, NC, United States). Before using in immune assays, PBMCs are pelleted at 453 g for 5 min and resuspended in 2 mL 10% FCS RPMI and lymphocytes counted using the trypan blue (diluted 1:1 with ddH_2_O, Corning, as above) membrane dye exclusion method. All cell counts are carried out by the same person to minimize variation.

##### 2.9.7.4. Mitogen stimulation of PBMC

The quantification of cytokine secretion by PMBCs stimulated with mitogens is used to evaluate the effector function of immune cells. Briefly, PBMCs are cultured in 2 mL 10% FCS RMPI-1640 medium (as above) for 48–72 h at 37°C and 5% CO_2_ without mitogen (unstimulated) or with mitogens. The tubes are set up to reach 1.00 × 10^6^ cells/mL. Depending on the cell recovery, in order of priority, we will stimulate PBMCs with (1) phytohemagglutinin (PHA) (25 μg/mL; Sigma-Aldrich, as above), (2) lipopolysaccharide (LPS) (5 μg/mL; Thermo Fisher Scientific, Carlsbad, CA, United States), (3) pokeweed (PWM) (55 μg/mL;), and (4) phorbol 12-myristate 13-acetate plus ionomycin (PMA + I) (2 μg/mL, Thermo Fisher Scientific, Carlsbad, CA, United States). After incubation, PBMCs are centrifuged at 428 g for 5 min at 18°C to pellet PBMCs. The supernatant is aliquoted and frozen at -80^o^ C for cytokine quantification. The post-pellet fraction is resuspended in 500 μL PBS, transferred to microtubes, and centrifuged at 906 g for 2 min. After discarding the supernatant, the pellet is stored at -80^o^ C for subsequent analysis. All assays are performed with the same number of cells per stimulation and the same concentration of mitogen. For the same participant in both time points we will use mitogens with the same lot number. The incubation time chosen for each mitogen was based off a concentration time course gradient.

##### 2.9.7.5. Quantification of *ex-vivo* cytokine secretion

Concentrations of IL-1β, IL-2, IL-6, IL-10, IFN-γ, and TNF-α in the supernatant are determined in duplicate by commercial ELISA kits (DuoSet^®^, R&D Systems, Minneapolis, MN, United States) according to the instructions of the manufacturer. The detection limits for the cytokines are as follows: IL-1β (250 to 3.91 pg/mL), IL-2 (1000–15.6 pg/mL), IL-6 (600–9.38 pg/mL), IL-10 (2000–31.3 pg/mL), IFN-γ (600–9.38 pg/mL), and TNF-α (1000–15.6 pg/mL). Cytokine concentrations are quantified using a microplate reader (Biotek^®^ Synergy™ H1, Agilent Technologies, Inc., Santa Clara, CA, United States), with an intra-assay coefficient of variation <10% and R^2^ of the standard curve ≥0.99. If samples are above the standard curve, they will be diluted onto the linear portion of the standard curve.

##### 2.9.7.6. Proliferation assay

The proliferation of T cells will be measured at baseline and after the intervention period using the fluorescence of a cell viability reagent (alamarBlue^®^ BUF012A, Bio-Rad Laboratories, Hercules, CA, United States). PBMCs are stimulated with anti-CD3 and anti-CD28 (BioLegend, as above). Briefly, black flat bottom 96-well cell culture plates (NUNC^®^, Thermo Fisher Scientific, Roskilde, Copenhagen, Denmark) are coated with 100 μL of 5 μg/mL anti-CD3 stock solution and are incubated overnight at 4°C. Then, stock solution is discarded using needle aspiration and rinsed twice with 200 μL sterile PBS. For the unstimulated and stimulated wells, 200 μL and 196 μL of 10% RPMI is added, respectively. The plate is set up to reach 1.00 × 10^6^ cells/mL in 200 μL. Samples are added in triplicate, with 4 μL of 50ug/mL anti-CD28 (previously diluted 10X in 10% RPMI) stimulation of coated CD3 wells. Plates are incubated at 37°C with 5% CO_2_ for 68 h. After 68 h, alamar blue is added equal to 10% of total volume (22.2 μL) and cells are incubated for 4 more hours for a total of 72 h. The plate is excited at 560 nm in a fluorescent microplate reader (Biotek^®^ Synergy™ H1, as above) and read at 590 nm. Data is expressed as arbitrary units/OD 590 nm, using individual values or delta.

##### 2.9.7.7. Fatty acids composition of total lipids

In conjunction with dietary information collected via the FFQ, circulating proportions of fatty acids in plasma and RBC will be assessed. Total lipids in plasma and RBCs (200 μL) are extracted using the Folch method with a 4:1 ratio (chloroform: methanol (2:1, 8 mL): 0.1 M potassium chloride (KCl, 1.8 mL) (79). Samples are vortexed then incubated overnight at 4°C. Samples are centrifuged at 906 g for 5 min with maximum brake and total lipids (bottom solvent layer) will be dried down under nitrogen gas and resuspended in boron trifluoride: hexane (1:1, 1.5 mL each) to methylate at 110°C for 1 hour. After cooling, 1 mL of ddH2O are added and samples will be incubated overnight at 4°C. Samples are centrifuged at 906 g for 5 min with maximum brake and total lipids (top layer) are dried down to be resuspended in 100 μL hexane for fatty acid methyl esters (FAME) proportional analysis, using an Agilent 8,890 gas chromatograph coupled with an autosampler (7693A) using a 100 m × 0.25 mm × 0.2um CP-Sil 88 fused capillary column for long chain fatty acids. Identification of fatty acids are based on external GLC 502 and GLC 37 standards. The following fatty acids proportion will be determined in the RBC membrane and plasma at baseline and after the intervention period: decanoic/capric (C10:0), undecanoic/undecylic (C11:0), dodecanoic/lauric (C12:0), tridecanoic/ tridecylic (C13:0), tetradecanoic/myristic (C14:0), tetradecenoic/myristoleic (C14:1 n-9), pentadecanoic/pentadecylic (C15:0), hexadecanoic/palmitic (C16:0), hexadecenoic/palmitoleic (C16:1 n-9), (9Z)-hexadec-9-enoic/palmitoleic (C16:1 n-7), heptadecanoic/margaric (C17:0), 10-heptadecenoic (C17:1), octadecanoic/stearic (C18:0), octadecenoic/elaidic (C18:1 T n-9), octadecenoic/oleic (C18:1 n-9), 11-octadecenoic acid/vaccenic (18:1 T n-7), octadecatrienoic /α-linolenic (C18:3 n-3), octadecadienoic/linoleic (C18:2 n-6), eicosanoic/arachidic (C20:0), 11-eicosenoic/gondoic (C20:1 n-9), 11,14-eicosadienoic (C20:2 n-6), 11,14,17-eicosatrienoic/dihomo-γlinolenic acid (C20:3 n-6), eicosatetraenoic/arachidonic (C20:4 n-6), eicosapentaenoic/EPA (C20:5 n-3), tetracosanoic/lignoceric (C24:0), tetracosenoic/nervonic (C24:1 n-9), docosapentaenoic/DPA (C22:5 n-3), docosahexaenoic/DHA (C22:6 n-3).

### 2.10. Statistical analysis

Continuous variables characterizing each study group will be reported as means with standard deviations or medians with interquartile ranges. Categorical variables will be represented as frequencies and proportions. Data will be checked for normality using the Shapiro–Wilk test, and logarithmic transformation will be employed for variables not normally distributed. The main comparisons in this study will be (1) between groups at each time point using analysis of variance with groups as the main effect followed by a multiple-comparison *post hoc* test and (2) within groups using paired *T*-test to assess differences between time points (i.e., baseline vs. post-intervention). Correlation analysis and linear regression models will be performed to verify the associations between outcomes (e.g., immune cell phenotypes and function, and systemic inflammation) and exposure (e.g., dietary intake or metabolic parameters, such as glucose, insulin, HOMA-IR, and HbA1c) variables. Mixed model repeated measures will be used to analyze variables of the OGTT to assess the effects of time, group, and time*group interaction. Overall comparisons of postprandial responses among groups will be determined using the incremental AUC with the trapezoid method. The statistical analyses will be adjusted for potential confounders and their interaction with the main group effect in cases where groups are unbalanced regarding factors such as physical activity level, use of medication (e.g., anti-diabetic drugs), and BMI, sex, and age if groups are not matched as initially planned. Sensitivity analyses will be conducted to verify the effect sizes of the primary outcomes of the study to assess the power and validity of our results. Comparative analyses using pertinent variables (e.g., age, sex, diet, metabolic profile, primary outcomes) will be performed between participants that completed the study with those who did not (i.e., participant dropouts or discontinuers). A *p*-value <0.05 will be considered to be statistically significant. A detailed analysis plan will be developed before the final analysis with a biostatistician.

### 2.11. Sample size

A total of 32 participants per group (*n* = 128) will provide adequate power (β = 0.81, α = 0.05) to assess a mean effect size of 30%, which was calculated based on differences in plasma CRP concentrations and *ex vivo* IL-2 secretion by PHA-stimulated PBMCs between Obese-NG and Obese-T2D participants. The sample size calculation was performed using data from a previous study (43) and the software G*Power. We applied a conservative attrition rate of 30% based on previous controlled feeding studies that ranged from 10–35% (81–85), totaling 166 participants.

### 2.12. Confidentiality, data management/monitoring and audit

Each participant will be assigned a unique study identifier for use on the case report forms, other trial documents, and the study database. All study records and documents will be treated as confidential and held securely in a locked filing cabinet. These will be retained for at least 5 years. The trial manager will make a separate confidential record of the name, date of birth, unique provincial health care number, phone number, email address, and participant screening number, to permit the identification of all participants enrolled in the study, in case additional follow-up is required. Only the trial manager and principal investigator will have access to the master list of the study that contains identifiable information. Study data are primarily managed using REDCap. All data from clinical visits, excluding data from unredacted source documents, will be uploaded to REDCap and securely maintained by the trial manager in electronic form. Medical information is available through the provincial health information system (Connect Care) and can be assessed by the appropriate medical personnel responsible for the participant’s healthcare. All manually entered data will be double-checked by a different member of the research team for accuracy. In addition, further verification of data will occur through the application of range to confirm if entered values are clinically possible values. Computer held data including the trial database will be held securely and password protected. Access to the study information will be limited to the trial staff and investigators. All collected data obtained as a result of this study is considered confidential and disclosure to third parties is only allowed when required by the ethics committee or regulatory authorities. Currently, there is no institutional quality assurance or scheduled audit procedures at the University of Alberta. Monitoring of the data is carried out by the trial manager as the data is collected at the HNRU. The trial manager is responsible to ensure completeness and accuracy of outcomes, adverse events, group allocations, informed consent, and completion/withdrawal. Access to the trial final dataset will be available to study investigators, trial manager, and graduate students. There are no contractual agreements that limit access to data.

### 2.13. Adverse event reporting

For this study, we followed the reporting requirements of the Health Research Ethics Board Biomedical Panel of the University of Alberta. Adverse events and other unintended effects will be collected weekly throughout the study from a self-monitoring form that participants will be asked to fill out daily. In addition to this method, participants will be advised to contact the study team directly to report any events. Expected events of this study, anticipated in the participants regardless of participation in research, may include unknown food allergies, gastrointestinal symptoms, changes in glycemia, and lipid profile. Although physiological changes may occur during participation in the study due to modifications in dietary patterns, they are not considered an adverse event unless they are unfavourable to the participant. To better understand if events could be related to the study we will evaluate the incident, its duration, and severity of symptoms. If a participant reports an incident of suspected foodborne illness we will investigate it to determine if there are other factors that may have caused it (i.e., improper food handling once food taken from HNRU, other illnesses in the household, food intolerance, etc.). Then we would send the food samples kept on site for testing to confirm if there was the presence of pathogens in the food. Only adverse events that are serious and unanticipated will be reported to the Research Ethics Office electronically through the Alberta Research Information Services (ARISE) System. Care will be provided to participants that suffer intervention-related harms. Participants will be instructed to seek medical attention and will be removed from the study if they are unable to comply with the feeding protocol due to acute illnesses not related to the study that may arise. If participants are willing to continue the feeding protocol after an acute illness (e.g., a cold), they will be asked to remain another week in the study to have their final appointments.

### 2.14. Protocol amendments

Protocol amendments, once approved by the research ethics board, were disseminated to investigator team by the research coordinator using direct communications and to trial participants, if pertinent. In addition, updates were posted at ClinicalTrials.gov.

### 2.15. Dissemination policy

The results of this study will be communicated within the scientific community through peer-reviewed publications, presentations at conferences attended by researchers and health professionals, and to the community at large, including coverage of study results in institutional websites and external media. A lay summary will be distributed via email to participants that agreed on communications on their last study appointment. We do not intend to use professional writers and manuscripts will be written by graduate students involved in the project. This trial does not have any publication restrictions. Authorship eligibility will be determined according to the recommendations of the International Committee of Medical Journal Editors guidelines.

## 3. Discussion

Obesity and T2D are associated with an increased risk of infections and a worse prognosis. Immune dysfunction in these conditions may be caused by abnormalities such as excess visceral adiposity (86), hyperglycemia (87), and unhealthy dietary habits (88). However, there is a lack of clinical trials that have controlled for diet and investigated immune function outcomes in males and females with obesity across different metabolic phenotypes.

To address this gap, the NutrIMM study aims to identify immune markers that are impaired in different health conditions, independent of the effect of diet. This study will also explore the relationship between dietary components, biological sex, and immune function. By understanding these differences, we hope to identify targeted and personalized approaches to improve immunity based on sex and metabolic phenotype.

Our investigation may also lead to the identification of circulating inflammatory markers that predict a lower immune function in individuals with obesity and T2D. This is important for diagnostic purposes as common techniques used to assess immune function are not practical for routine diagnosis. Moreover, our study may identify key nutrients that could improve immune function and guide future targeted and personalized interventions for subsequent trials.

The NutrIMM study is the first to determine the independent contribution of diet, sex, excess body fat, and altered glucose levels on inflammation and immune dysfunction associated with obesity. Other strengths of our protocol include assessing immune function outcomes under controlled feeding conditions, which provides valuable information about the effects of different foods, nutrients, and dietary patterns on health outcomes. This type of study allows for careful monitoring and manipulation of food intake, direct measures of physiological responses to different foods and nutrients, and the development of personalized nutrition recommendations based on an individual’s unique physiological response (89–91).

However, the limitations of dietary clinical trials include limited generalizability to larger populations or diverse demographic groups and the inability to accurately reflect real-world dietary patterns that can vary on a daily basis (92). Feeding studies also have a limited application in long-term follow-up to assess the effects of dietary interventions on chronic disease risk or other long-term health outcomes (93). Finally, such studies can be expensive and time-consuming to conduct, which limits the number of participants and studies that can be done and hence the generalizability of findings (94).

## Trial status

As of June 16, 2023, the NutrIMM protocol version is 2.1. Enrollment of participants started in October 2019 and will continue until the desired sample size is achieved. Recruitment is expected to be completed in July 2023.

## Author’s note

Participants will receive a $100 CAD gift card to a grocery store as token of appreciation for their participation upon completion of the study. Parking and/or public transportation expenses will be offered for the costs incurred for study appointments.

## Data availability statement

The original contributions presented in the study are included in the article/Supplementary material, further inquiries can be directed to the corresponding author.

## Ethics statement

The studies involving humans were approved by Research Ethics Board 3: Health Research Ethics Board-Health Panel/ University of Alberta. The studies were conducted in accordance with the local legislation and institutional requirements. The participants provided their written informed consent to participate in this study.

## Author contributions

JT: conceptualization, methodology, formal analysis, investigation, writing – original draft, visualization, supervision, and project administration. MS: methodology, formal analysis, writing – review and editing, investigation, visualization, and supervision. AM: methodology, formal analysis, validation, writing – review and editing, investigation, and supervision. PC: investigation, writing – review and editing, supervision, and project administration. CR: conceptualization, methodology, writing – review and editing, supervision, and funding acquisition. All authors contributed to the article and approved the submitted version.

## Funding

Financial support for NutrIMM was provided by the Canadian Institutes of Health Research (grant number 398909). The funding agency did not have any role in the study design, collection, analysis, interpretation of data, writing the manuscript or decision to submit the manuscript.

## Sponsor

The NutrIMM study is an investigator-initiated trial, therefore the principal investigator (CR) is the trial sponsor. The address of the sponsor is 4-002G Li Ka Shing Centre, Edmonton, Alberta, T6G 2E6, Canada. Telephone: +1 (780) 248–1827. Email: cr5@ualberta.ca. The sponsor-investigator is responsible for designing the study, overseeing data collection, study management, data analysis and interpretation, manuscript writing, dissemination of results, and has ultimate authority over all these activities.

## Conflict of interest

The authors declare that the research was conducted in the absence of any commercial or financial relationships that could be construed as a potential conflict of interest.

## Publisher’s note

All claims expressed in this article are solely those of the authors and do not necessarily represent those of their affiliated organizations, or those of the publisher, the editors and the reviewers. Any product that may be evaluated in this article, or claim that may be made by its manufacturer, is not guaranteed or endorsed by the publisher.

## Footnotes

^1^ nutrimm.ualberta.ca
