# Corrigendum: The nutrition and immunity (nutrIMM) study: protocol for a non-randomized, four-arm parallel-group, controlled feeding trial investigating immune function in obesity and type 2 diabetes

**DOI:** 10.3389/fnut.2023.1304095

**Published:** 2023-10-11

**Authors:** Jenneffer Rayane Braga Tibaes, Maria Inês Barreto Silva, Alexander Makarowski, Paulina Blanco Cervantes, Caroline Richard

**Affiliations:** ^1^Department of Agricultural, Food and Nutritional Science, University of Alberta, Edmonton, AB, Canada; ^2^Department of Applied Nutrition, Rio de Janeiro State University, Rio de Janeiro, Brazil; ^3^Department of Medical Microbiology and Immunology, University of Alberta, Edmonton, AB, Canada

**Keywords:** immunology, hyperglycemia, nutrition, obesity, type 2 diabetes, insulin resistance, North American diet

In the published article, there was an error in the **Acknowledgments** statement. Support received by CR from the Canada Research Chair program was omitted from the statement.

The corrected Acknowledgments statement appears below.

## Acknowledgments

We would like to thank all research participants, the staff from the HNRU, volunteers, undergraduate and summer students that contributed to this project. Experiments are performed at the University of Alberta Faculty of Medicine and Dentistry Flow Cytometry Facility (RRID:SCR_019195) and Lipidomics Core (RRID:SCR_019176), which receive financial support from the Faculty of Medicine and Dentistry and Canada Foundation for Innovation (CFI), and the latter also from the Natural Sciences and Engineering Research Council of Canada (NSERC) awards to contributing investigators. CR is supported by the Canada Research Chair program (CRC-2018-00081).

The authors apologize for this error and state that this does not change the scientific conclusions of the article in any way. The original article has been updated.

