# Supplementary material for: The nutrition and immunity (nutrIMM) study: protocol for a non-randomized, four-arm parallel-group, controlled feeding trial investigating immune function in obesity and type 2 diabetes
Source: Front Nutr. 2023 Sep 1;10:1243359. doi: 10.3389/fnut.2023.1243359 (PMC10505731; doi:10.3389/fnut.2023.1243359)
Supplement: Supplementary file 1 [file Table_1.docx]

Supplementary Material

The Nutrition and Immunity (nutrIMM) Study: Protocol for a non-randomized, four-arm parallel-group, controlled feeding trial investigating immune function in obesity and type 2 diabetes

Jenneffer Rayane Braga Tibaes^1^, Maria Inês Barreto Silva^1,2^, Alexander Makarowski^1^, Paulina Blanco Cervantes^1^, Caroline Richard^1,3*^

^1^Department of Agricultural, Food and Nutritional Science, University of Alberta, Edmonton, Alberta, Canada

^2^Department of Applied Nutrition, Rio de Janeiro State University, Rio de Janeiro, Brazil

^3^Department of Medical Microbiology and Immunology, University of Alberta, Edmonton, Alberta, Canada

*** Correspondence:**Caroline Richard
cr5@ualberta.ca

**Supplementary Table 1.** Antibody conjugates and staining reagents for flow cytometry

| **Specificity^1^** | **Fluorophore** | **Clone** | **Dilution** | **Purpose** | **Panel #** |
| --- | --- | --- | --- | --- | --- |
| CD3 | BV421 | OKT3 | 1:20 | Chain of the TCR complex, T cell | 1, 2, 7, 8 |
| CD4 | PerCP | OKT4 | 1:20 | Ligand of MHCII, T_H_ cell | 1, 2, 3 |
| CD4 | BV510 | OKT4 | 1:20 | Ligand of MHCII, T_H_ cell | 8 |
| CD8 | FITC | SK1 | 1:20 | Co-receptor with MHCI, T_C_ cell | 2, 3 |
| CD45RA | APC | HL100 | 1:20 | T cell naïve | 2 |
| CD45RO | BV711 | UCHL1 | 1:50 | T cell memory | 2 |
| CD25 | PE | M-A251 | 1:50 | Low affinity IL-2 receptor α chain, T_reg_ cell, activation marker | 1, 3 |
| FOXP3 | AF647 | 150D | 1:10 | Regulatory gene, T_reg_ cell | 1 |
| CD28 | BV711 | CD28.2 | 1:20 | Co-stimulatory molecule, T cell | 3 |
| CD152 (CTLA-4) | BV421 | BNL3 | 1:7 | Inhibitory signal, T_H_ cell/T_reg_ cell | 3 |
| CD192 (CCR2) | PE-Cy7 | K036C2 | 1:100 | Ligand to MCP 1-4 | 3, 4 |
| CD183 (CXCR3) | FITC | G025H7 | 1:20 | CR (CXCL9, CXCL10, CXCL11), T_H_ cell | 8 |
| CD185 (CXCR5) | PE-Cy7 | J252D4 | 1:50 | CR (CXCL13), T_H_ cell | 8 |
| CD194 | PE | L291H4 | 1:20 | CR (CCL17, CCl22), T_H_ cell | 8 |
| CD196 (CCR6) | BV711 | G034E3 | 1:20 | CR (CCL20), T_H_ cell | 8 |
| CCR10 | APC | 6588-5 | 1:10 | CR (CCL27), T_H_ cell | 8 |
| CD19 | APC | 4G7 | 1:10 | Transmembrane glycoprotein, B cell | 4 |
| CD20^a^ | PE | 2H7 | 1:100 | Transmembrane spanning protein, B cell | 4 |
| CD54 (ICAM-1) | FITC | HA58 | 1:10 | Cellular adhesion | 4, 5 |
| CD80 | BV421 | 2D10 | 1:10 | Activation marker | 4, 5, 6 |
| CD11c | PE | 3.9 | 1:100 | Transmembrane glycoprotein, DC | 5, 6 |
| CD14 | PerCP | M5E2 | 1:10 | LPS receptor, monocytes | 5 |
| CD86 | PE-Cy7 | BU63 | 1:50 | Activation marker | 5 |
| CD273 | APC | 24F.10C12 | 1:10 | Co-stimulatory/inhibitory, DC | 5, 6 |
| HLA-DR | BV711 | L243 | 1:20 | MHC II isotype, antigen presentation, DC, monocytes | 5, 6 |
| CD123 | FITC | 6H6 | 1:10 | Transmembrane α chain of the IL-3 receptor, DC | 6 |
| CD56 (NCAM) | FITC | HCD56 | 1:10 | Transmembrane glycoprotein, NK cell | 7 |
| CD16 | PE | 3G8 | 1:200 | Low affinity IgG receptor III, NK cell | 7 |

AF, alexa fluor; APC, allophycocyanin; BV, brilliant violet; DC, dendritic cell; FITC, fluorescein isothiocyanate; NK, natural killer; PE, phycoerythrin; T_C_, cytotoxic T cell. PE-Cy7, phycoerythrin-cyanide7; PerCP, peridin-chlorophyll-protein; T_H_, helper T cell; T_reg_, regulatory T cell. ^1^All antibodies were manufactured by BioLegend (San Diego, CA, USA), unless otherwise stated. ^a^ Cell Signaling Technology (Danvers, MA, USA).
