# Supplementary material for: The nutrition and immunity (nutrIMM) study: protocol for a non-randomized, four-arm parallel-group, controlled feeding trial investigating immune function in obesity and type 2 diabetes
Source: Front Nutr. 2023 Sep 1;10:1243359. doi: 10.3389/fnut.2023.1243359 (PMC10505731; doi:10.3389/fnut.2023.1243359)
Supplement: Supplementary file 2 [file Data_Sheet_1.ZIP › Supplementary File 1.pdf]

UNIVERSITY OF ALBERTA

DEPARTMENT OF AGRICULTURAL, FOOD AND NUTRITIONAL SCIENCE
FACULTY OF AGRICULTURAL, LIFE & ENVIRONMENTAL SCIENCES

Division of Human Nutrition
4-002F Li Ka Shing Centre for Health Research
Edmonton, Alberta, Canada T6G 2P5
Tel: 780.492.8960
Fax: 780.492.9270
www.afns.ualberta.ca

PARTICIPANT CONSENT FORM

Title of Study: The Alberta NutrIMM Study (Nutrition and Immunity study)

Principal Investigator: Dr. Caroline Richard, [redacted]
Research/Study Coordinator: Paulina Blanco, MSc [redacted]

Why am I being asked to take part in this research study? Excess weight, nutrition, and blood sugar levels can all affect immune function, which in turn can affect your risk for heart disease and type 2 diabetes (T2DM). It is not known how diet, blood sugar, and weight affect immune function.

This form contains information about the study. Before you read it, a member of the study team will explain the study to you in detail. You are free to ask questions if there is anything you do not understand. You will be given a copy of this form for your records.

What is the reason for doing the study? The purpose of the study is to look at how weight, diet and blood sugar levels affect immune function. Results of the study will be compared to results of the control group (which will go through the same activities as the experimental groups).

What will happen in the study? Based on your blood results, you will be assigned to one of four study groups: participants with normal blood sugar levels (NG; control group); higher weight participants with normal blood sugar levels; higher weight participants who have pre-diabetes; higher weight participants who have type two diabetes. Participants of all four groups will consume a typical North American/Canadian diet for 4 weeks.

Figure 1: Summary of timeline and participant activities at each stage of the study:

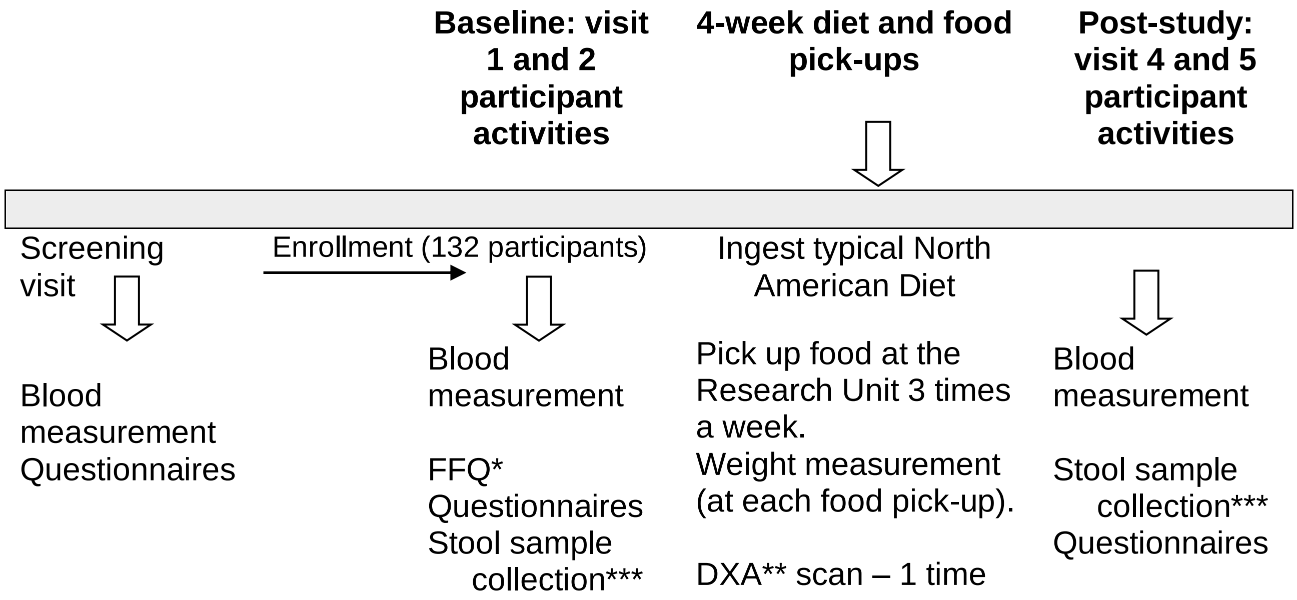

*FFQ = food frequency questionnaire
**DXA = Dual Energy X-ray Absorptiometry measurement
***Part of stool sub-study (which requires separate consent form)

Baseline: Visit Day 1 and Day 2: Estimated time is 45-60 minutes on each visit day. Fasted blood measurements will be done on two consecutive days (by a trained phlebotomist – someone who is specially trained in taking blood). Blood sugar, lipid panel (cholesterol), C -reactive protein (CRP), Insulin, HbA1c, CBC (complete blood count) and differential, and immune levels will be measured in these blood samples. Depending on the time from screening to baseline we might also need to measure your weight, waist size and blood pressure. To get a good snapshot of what you usually eat, we will also give you instructions on how to fill out a food frequency questionnaire (from home). Lastly, you will need to fill out a physical activity and background questionnaire and collect a stool sample (if you are part of the stool sub-study) before study start.

4-week food consumption and food pick-ups: You will eat a typical North American diet for 4 weeks to maintain your body weight. You will need to pick up your home cooked meals around three times a week at the HNRU, where we will also measure your weight (which will take about 10 minutes). You will take your weekend meals home on Fridays. You will need to bring all your food home and refrigerate it as soon as possible (maximum 1-hour window). The Study Coordinator will give you instructions on beverages other than water, outside of the study, that you can drink.

One time during the 4 weeks you will have your body fat analyzed using Dual Energy X-ray Absorptiometry (DXA). The purpose of the DXA scan is to assess body composition by quantifying bone, muscle and fat mass. This is a painless, non-invasive test. You will have to put on a hospital gown and lay on an x-ray bed. The scan takes 5 minutes. No special preparation is necessary. Women who participate in the study will have to complete a pregnancy test prior to the test to confirm you are not pregnant.

Post-study: Visit Day 1 and Day 2: Estimated visit time: for visit post-study visit Day 1, visit time is about 60 minutes and on Day 2, it is 3.5 to 4 hours. In post-study visit Day 1, you will have fasted blood taken (to look at lipid panel (cholesterol), blood sugar, CRP, Insulin, HbA1c and CBCD and the immune system).
In post- study visit (Day 1), we will also need to measure your weight, blood pressure and waist/hip circumference. In visit on Day 2, we will do an oral glucose tolerance test to measure blood sugar levels, which involves taking blood every 30 minutes for 3.5-4 hours after inserting an IV by a trained phlebotomist, after you drink a sugary beverage. Lastly, you will need to collect a stool sample and bring it back to us to us at either of your final visits.

What are the risks and discomforts? The study requires 4 blood draws and one IV. A needle will be inserted into a vein and blood will be withdrawn for lab tests by a trained phlebotomist. It is possible that you may experience mild pain, fainting, bleeding, bruising, and/or an infection at the insertion site. Bruising is common, but usually goes away after a few days. Infection, dizziness, and fainting are rare during this procedure. If you have a pacemaker or another electrical device in your body, you should not have a blood measurement because the blood measurement may prevent the device from working properly.

There is also a small possibility that you may experience an allergic reaction to a food (if you have a food allergy). Although, every effort will be made to reduce participant exposure to the food that they are allergic to, if exposure does occur, an epi-pen will be kept in the HNRU, and medical attention will be sought out (if needed). However, we cannot guarantee foods will not be in contact with priority allergens, so if there are any food allergies of your concern, you must need to be aware of this.

The X-ray dose associated with a total body scan is very low and safe for repeated measurements. With the exception of pregnant women, there are no known risks associated with DXA scans. Having a DXA scan does not make it unsafe for you to have other x-rays taken in the near future. You may ask the technologist to stop the test at any time without jeopardy to you. All information collected from your data base will be stored using your study identification study not your name.

There may be risks in this study that are currently not known. If we find out anything new during the course of this research which may change your willingness to be in the study, we will tell you about these findings.

Risk of exposure to COVID-19 with your participation includes exposure to others (research personnel and other participants) and increased time within our research unit. Measures undertaken to reduce this risk include ensuring all personnel

and participants wear a mask, frequent hand washing (or use of hand-sanitizers) and limiting the unit capacity in accordance to campus guidelines. All hard surfaces and common touched areas are disinfected before and after each visit. Staff and participants will be screened for symptoms consistent with COVID-19, recent travels, and contact with others who are suspected or test positive for COVID-19. Additionally, all of the menu preparations will be done wearing a mask. If you become sick, or have any symptoms of COVID-19, you must inform study staff.

What are the benefits to me? There are no direct benefits to participants. Through participation in this study, you will also contribute to the advancement of knowledge about how weight, blood sugar, and nutrition affect immunity. You will also receive meals for 4 weeks.

What will I need to do while I am in the study? For all of the blood measurements, you need to make sure that you don't have anything to eat or drink (except water) 9 hours before the measurement. Also, during the 4-week diet period, you need to eat only foods prepared by the study team for 4 weeks. The study coordinator will give you information about other beverages that you can drink outside of the study. You will need to record any food/drinks eaten or drank outside of the study in a food journal, given you to you by the study coordinator at the beginning of the study. Lastly, you will need to maintain your regular physical activity levels throughout the 4-week diet period. You will be also asked to follow food safety instructions of food handling and transportation. This is to ensure that the food provided to you remains safe to eat.

Do I have to take part in the study? Being in this study is your choice. If you decide to be in the study, you can change your mind and stop being in the study at any time, and it will in no way affect the care or treatment that you are entitled to.

Can my participation in the study end early? The research coordinator may withdraw you from this study if you don't show up for your study visits without previous notice for rescheduling or if she feels that it would be in your best medical interests to withdraw from the study. We will be assessing compliance to the diet on a weekly basis and if the overall compliance below 90% is observed (including food consumed in addition to the menu), participants will be excluded before the end of the study. Incentives will be given only to participants who successfully complete their participation in the study.

Will I be paid to be in the research? You will not be paid for participating in this study. However, to thank you for your time with the overall study, you will receive a $100 grocery store gift card upon completion of the study. Your parking and public transit costs will be covered for participating at the end of the study with additional grocery store gift card(s). We can only cover a limit of $100 maximum for parking and/or public transit expenses. If your expenses are less than $100, we reimburse closer to the amount spent (rounded). You need to keep and provide all your receipts as proof of your expenses. The $100 gift card (token of appreciation) is only provided

upon completion of the study. However, if you end your participation earlier, your parking/ public transit expenses will be covered by the total amount of when you stop your participation.

Privacy and Confidentiality
During this study we will be collecting information (or “study data”) about you. We will use the data to help answer research questions and we will share (or “disclose”) your information with others such as the study sponsor and other researchers. Your study data may also be shared with government departments involved in the approval of drugs for sale in a country. These departments are often called “regulatory authorities”. An example of a regulatory authority is Health Canada.

Below we describe in more detail how your data will be collected, stored, used and disclosed.

What data will be collected? During this study we will be collecting data about you. Examples of the types of data we may collect includes your name, where you live, your ethnic background, your date of birth, your age, your health conditions, your health history, your medications and results of tests or procedures that you may have had. We will only look for and collect the information that we need do the research. We will get this information by asking you questions and doing the tests outlined in this form. We will also look at your medical chart (paper or electronic) held by the study doctor or other doctors you have seen (such as your family doctor).

How will the study data be stored? The study data we collect which will include your name will be securely stored by the study coordinator during and after the study. We will also put a copy of this consent form in your clinical record, so that doctors you see in the future will know you were in the study. In Canada, the law says we have to keep the study data stored for at least 15 years after the end of the study.

The study doctor/coordinator will not release your name to anyone unless the law says that they have to.

How will the study data be used?
Your study data will be coded (with a number) so that it no longer contains your name, address or anything else that could identify you. Only the study coordinator and study doctor will be able to link your coded study data to you.

Who will be able to look at my health data?

During research studies it is important that the data we get is accurate. Therefore, your study data and original medical records may also be looked at by people from: the study sponsor, the University of Alberta auditors and members of the Research Ethics Board.

By signing this consent form you are saying it is ok for the study doctor/staff to collect, use and disclose information from your medical records and your study data as described above. More specifically, by signing this consent form you are authorizing Alberta Health Services (AHS) to allow the research team access to your individually identifying health information for research purposes (blood tests ordered by us through AHS and blood work results).

If you would like to see the study data collected about you, please ask the study doctor/coordinator. You will be able to look at the study data about you and you can ask for any mistakes to be corrected. The study doctor/coordinator may not be able to show you your study data right away and you may have to wait until the study is completed or another time in the future before you can see your study data.

If you leave the study, we will not collect new health information about you, but we will need to keep the data that we have already collected.

What if I have questions?
If you have any questions about the research now or later, please contact **Dr. Caroline Richard (780-248-1827) or Paulina Blanco (780-492-9506)**. If you suffer a research related injury – please contact either Investigator listed at their respective phone number.

If you have any questions regarding your rights as a research participant, you may contact the Health Research Ethics Board at 780-492-2615. This office is independent of the study investigators.

The study is being conducted/sponsored by the Canadian Institutes of Health Research (CIHR). The Institution and study staff are getting money from the study sponsor to cover the costs of doing this study. You are entitled to request any details concerning this compensation from the Principal Investigator.

How do I indicate my agreement to be in this study?

By signing below, you understand:

- That you have read the above information and have had anything that you do not understand explained to you to your satisfaction
- That you will be taking part in a research study
- That you may freely leave the research study at any time
- That you do not waive your legal rights by being in the study
- That the legal and professional obligations of the investigators and involved institutions are not changed by your taking part in this study.

SIGNATURE OF STUDY PARTICIPANT

____
Signature of Participant

________________________________ _____________________
Name of Participant Date

SIGNATURE OF PERSON OBTAINING CONSENT

____
Signature of Person Obtaining Consent

________________________________ _____________________
Name of Person Obtaining Consent Date

SIGNATURE OF THE WITNESS

____
Signature of Witness

________________________________ _____________________
Name of Witness Date

A signed copy of this consent form has been given to you to keep for your records and reference.

UNIVERSITY OF ALBERTA

Variance Sub-Study – Informed Consent Form

Project Title: Gut Microbiome sub-study of the NutrIMM Study
Department: Agricultural, Food and Nutritional Science [AFNS]

Investigator	Position	Phone Number	E-mail Address
Dr. Caroline Richard	Assistant Professor<br>Study Coordinator	[redacted]	[redacted]

Before beginning this research study, you signed an Information & Consent Form for the main study describing the study and your rights as a study participant. All of these same rights apply to this consent.

You are being asked to participate in an optional sub-study (which is separate from the main study) to assess how the composition and function of a person's gut bacteria fluctuates over the course of 4 weeks while eating a typical North American/Canadian diet for weight maintenance.

Background
Weight and high blood sugar levels have been linked to low-grade inflammation throughout the body. This inflammation is thought to contribute to diseases like heart disease and diabetes. The microbes (bacteria) in our gut are thought to be a link between weight, high blood sugar levels, and this inflammation where certain levels of weight and blood sugar values may be associated with higher levels of inflammation based on the type of bacteria that are present in the gut.

Diet is another way that we can change the type of microbes in our gut. We know that fiber, sugar and other dietary components have a big impact on our gut microbes. By changing our diet, we can change the microbes in our gut, which may improve inflammation.

What is the purpose of the study?
The purpose of this study is to determine how a typical North American/Canadian diet affects the gut microbes in relation to weight and blood sugar levels. This in turn will help define the role of the type of bacteria that live in our gut in providing health benefits.

What is involved in the Stool Sub-Study?
In the stool sub-study, you will do exactly what is described in the main study, except that a stool sample will be collected before and after the 4 week dietary period (as part of the stool sub-study). Stool samples must be dropped off +/- 1 day of when they are due. The type of bacteria in stool will be analyzed and stored at a later date, and will not be part of the main study data analysis.

Will I be paid if I Participate?
You will not be paid to participate in the gut-microbiome sub-study.

CONSENT

I understand that all other items from main consent form apply. I understand what is involved in the gut microbiome sub-study and agree to participate.

________________________________ ________________________________

Name (Please print) Signature

____

Date

I believe the person signing this form understands what is involved in sub-study and voluntarily agrees to participate.

________________________________ ________________________________

Signature of Investigator Date
