# Supplementary material for: The nutrition and immunity (nutrIMM) study: protocol for a non-randomized, four-arm parallel-group, controlled feeding trial investigating immune function in obesity and type 2 diabetes
Source: Front Nutr. 2023 Sep 1;10:1243359. doi: 10.3389/fnut.2023.1243359 (PMC10505731; doi:10.3389/fnut.2023.1243359)
Supplement: Supplementary file 2 [file Data_Sheet_1.ZIP › Supplementary File 2.pdf]

NutrIMM Study Pre-Screening Survey

The following questionnaire will be used help us to determine your eligibility to participate in the NUTRIMM Study. This questionnaire will take approximately 2-5 minutes to complete. All responses to questions will only be used to ensure that you are eligible and that it is safe for you to participate in our study. Only study staff will be able to see your answers.

At the bottom of this questionnaire you can find the information letter with all the details of the NutrIMM study. You can also access this information on our website: nutrimm.ualberta.ca

By completing this questionnaire, you understand:That you are being screened in order to take part in a research study.That you may choose not to answer any questions and may freely exit out of the questionnaire at any time.That you will be contacted by study staff via email or telephone to inform about your eligibility.If you seem eligible, we will invite you to come to the Human Nutrition Research unit for about 45-60 minutes. At this visit, we will get some background information from you, measure your blood pressure/weight/height/and waist size, and do a blood draw.
Thank you so much for your interest in the NutrIMM Study!

Name

____
(Please, provide first and last name)

What is your email address?

____
(Please, provide the best email to contact you back at)

What is your phone number?

____
(###-###-####)

What is your biological sex at birth?

- ◯ Male
- ◯ Female
- ◯ Other

Do you have a regular menstrual cycle?

- ◯ Yes
- ◯ No (please provide details in the next question)
- ◯ Not applicable, I am menopausal
- ◯ Not applicable, I am post-menopausal

Please provide details about your menstrual cycle irregularity (e.g. IUD, hormonal contraceptives, surgery, PCOS, etc).

____

Are you pregnant or breastfeeding?

- ◯ Yes
- ◯ No

How old are you?

____

What unit are you reporting your height in?

- ◯ centimeters (cm)
- ◯ meters (m)
- ◯ feet/inches (ft/in)

Please, inform your height in centimeters

____
(###.# centimeters (cm))

Please, inform your height in meters	______________________ (#.## meters (m))
What is your height (feet)?	______________________ (# feet (ft))
Inches	______________________ (# inches (in))
Height in Meters - Hidden Survey	______________________
What unit are you reporting your weight in?	◯ pounds (lbs)<br>◯ kilograms (kg)
Please, inform your weight in pounds	______________________ (#.# pounds (lb))
Please, inform your weight in kilograms	______________________ (#.# kilogram (kg))
Weight in KG	______________________
BMI - Hidden Survey	______________________
Has your weight been stable for the past 6 months? (Within 3% or if you do not have a scale, do your clothes fit the same?)	◯ Yes<br>◯ No
Do you have type 2 diabetes?	◯ Yes<br>◯ No
Were you diagnosed with type 2 diabetes more than 6 months ago?	◯ Yes<br>◯ No
Are your blood sugar levels stable?	◯ Yes<br>◯ No
Do you have a history of any disease(s) or health problem(s) other than type 2 diabetes?<br><br>(Example: cardiovascular disease, renal disorders, high cholesterol levels, PCOS, or the presence of a disorder involving hormone problems, etc.)	◯ Yes<br>◯ No
Please, provide brief details of your condition(s).	______________________

Do you regularly take aspirin, Ibuprofen, antihistamines, fish oils, omega-3 supplements or any other supplements regularly (eg. vitamins and minerals)?	◯ Yes<br>◯ No
Are you taking any lipid lowering medications?	◯ Yes<br>◯ No
Do you smoke (tobacco, Marijuana, e-cigarettes)?	◯ Yes<br>◯ No
Are you vegetarian?	◯ Yes<br>◯ No
Do you have any dietary restrictions (e.g. food allergies, intolerances, or personal/religious related)?	________________________________<br>(Please, list here if any)
How did you hear about our study?	________________________________
How did you hear about our study (choose all that apply)?	☐ Flyer at the University<br>☐ Word of mouth<br>☐ Facebook or Instagram page<br>☐ Facebook or Instagram advertising<br>☐ Other
Please, specify "Other"	________________________________

Study Information Sheet

[Attachment: "Information and Consent NUTRIMM STUDY.pdf"]
