# Supplementary material for: The nutrition and immunity (nutrIMM) study: protocol for a non-randomized, four-arm parallel-group, controlled feeding trial investigating immune function in obesity and type 2 diabetes
Source: Front Nutr. 2023 Sep 1;10:1243359. doi: 10.3389/fnut.2023.1243359 (PMC10505731; doi:10.3389/fnut.2023.1243359)
Supplement: Supplementary file 2 [file Data_Sheet_1.ZIP › Supplementary File 4.pdf]

nutrIMM Menu

	Monday<br>Day 1	Tuesday<br>Day 2	Wednesday<br>Day 3	Thursday<br>Day 4	Friday<br>Day 5	Saturday<br>Day 6	Sunday<br>Day 7
**Breakfast**	French Toast<br>Maple Syrup<br>Canned Peaches	Toast<br>Butter<br>Egg (boiled)	Cereal<br>Milk	Veggie Omelet<br>Toast<br>Butter	Yogurt<br>Granola	Pancakes<br>Butter<br>Sausage<br>Syrup	Rice Krispies<br>Milk
**Lunch**	Pizza<br>Potato Chips<br>Chocolate Chip Muffin	Ham Sandwich<br>Blueberry Muffin	Hamburger Hash<br>Yogurt	Sweet and Spicy<br>Chicken Wrap<br>Chocolate Muffin	Pork Stirfry with Rice<br>Carrots	Chili<br>Bun<br>Margarine<br>Chocolate Chip<br>Muffin	Beef Taco<br>Chocolate Chip<br>Muffin
**Dinner**	Pot Roast<br>Veggies<br>Brownie	Fettuccine Alfredo<br>with Chicken<br>Potato Chips	Spaghetti with Beef<br>Chocolate Chip<br>Cookie	Thai Peanut Rice Bowl<br>Shortbread Cookie	Beef Lasagna<br>Blueberry Muffin	Meatloaf<br>Potatoes<br>Carrots<br>Chocolate Chip<br>Cookie	Hamburger Helper<br>Green Beans<br>Brownie

Note: The quantity of food is adapted to the calories that each participant needs with the purpose of maintaining the same weight throughout the 4 weeks.
