# Supplementary material for: The nutrition and immunity (nutrIMM) study: protocol for a non-randomized, four-arm parallel-group, controlled feeding trial investigating immune function in obesity and type 2 diabetes
Source: Front Nutr. 2023 Sep 1;10:1243359. doi: 10.3389/fnut.2023.1243359 (PMC10505731; doi:10.3389/fnut.2023.1243359)
Supplement: Supplementary file 2 [file Data_Sheet_1.ZIP › Supplementary File 5.pdf]

Menu and Health Tracker

Date: ____________ **Week**: ____________ **Participant ID**: ____________

INSTRUCTIONS

Please check the circle of each item that was fully consumed. For items you were not able to eat the entire portion, please try to eat equal portions of each component. Report how much was actually consumed in the notes section. Please report any extra food or drink items you ate, if any, in the notes section. **All food items with the word "hot" MUST be heated properly at a high temperature to ensure continuous food safety. If heated on microwave, food needs to be stirred halfway to ensure equal heat on the food.**

Day	Meal	Time	Location	Food	Check if consumed	Notes
**TUE**	Breakfast			Toast<br>Butter<br>Eggs (hot)	○<br>○<br>○	
	Lunch			Ham Sandwich<br>Blueberry Muffin	○<br>○	
	Dinner			Fettucine with Chicken (hot)<br>Potato Chips	○<br>○	
**WED**	Breakfast			Cheerios<br>Milk	○<br>○	
	Lunch			Hamburger Hash (hot)<br>Yogurt	○<br>○	
	Dinner			Spaghetti with Beef (hot)<br>Chocolate Cookie	○<br>○	
**THU**	Breakfast			Veggie omelet (hot)<br>Toast<br>Butter	○<br>○<br>○	
	Lunch			Sweet and Spicy Chicken (hot)<br>Wrap<br>Chocolate Muffin	○<br>○	
	Dinner			Thai Peanut Chicken (hot)<br>Rice<br>Shortbread Cookie	○<br>○<br>○	
**FRI**	Breakfast			Yogurt<br>Granola	○<br>○	
	Lunch			Pork Stirfry (hot)<br>Rice<br>Carrots<br>Dressing	○<br>○<br>○<br>○	
	Dinner			Lasagna (hot)<br>Blueberry Muffin	○<br>○	

Menu and Health Tracker

**SAT**	Breakfast			Pancakes<br>Maple Syrup<br>Sausage (hot)<br>Butter	○<br>○<br>○<br>○	
	Lunch			Chili (hot)<br>Bun<br>Margarine<br>Chocolate Muffin	○<br>○<br>○<br>○	
	Dinner			Meatloaf (hot)<br>Potatoes<br>Carrots<br>Chocolate Cookie	○<br>○<br>○<br>○	
**SUN**	Breakfast			Rice Krispies<br>Milk	○<br>○	
	Lunch			Beef Taco (hot)<br>Chocolate Muffin	○<br>○	
	Dinner			Hamburger Helper (hot)<br>Green Beans<br>Brownie	○<br>○<br>○	
**MON**	Breakfast			French Toast (hot)<br>Peaches<br>Maple Syrup	○<br>○<br>○	
	Lunch			Pizza (hot)<br>Potato Chips<br>Chocolate Muffin	○<br>○<br>○	
	Dinner			Pot Roast (hot)<br>Veggies<br>Brownie	○<br>○<br>○	

Weekly health status check:

Did you have any symptoms this week? ○ Yes ○ No<br>If yes, please write **when** and **what symptom(s):**	Did you take any medications this week? ○ Yes ○ No<br>If yes, please write **when** and what **medication(s)**:

Version: December 2021
